# Exploring an e-learning community’s response to the language and terminology use in autism from two massive open online courses on autism education and technology use

**DOI:** 10.1177/1362361320987963

**Published:** 2021-02-11

**Authors:** Jiedi Lei, Lauren Jones, Mark Brosnan

**Affiliations:** 1 University of Bath, UK; 2 King’s College London, UK

**Keywords:** autism, disability, identity-first, neurodiversity, person-first, quality of life, terminology

## Abstract

Prior research has identified the divergence across different stakeholder groups in the semantic choice of language when describing autism, as members of the autism and autistic community preferred to use *identity*-*first* language (autistic person), whereas professionals were more likely to use *person*-*first* language (person with autism). This study explored 803 e-learners’ responses from their comments across two massive open online courses on autism education held between 2017 and 2019. Comments from members of the autistic and autism community and professionals were analysed together using thematic analysis, to identify shared opinions on what, why and how language should be used when describing autism across stakeholder groups. Learners agreed that autistic individuals should guide others on which terminology to use when describing autism and that the diagnostic label is a way to facilitate understanding across stakeholder groups and help the individual gain access to support. Semantic language choices may matter less as long as the person’s difficulties are clearly acknowledged, with adaptations made to meet their specific needs. Adding to a growing body of literature on terminology use in autism research and practice, we highlight that consideration for semantic choice of language use should focus on communicating an individual’s strengths and differences.

**Lay abstract:**

Within the neurodiversity movement, one recent divergence is in the semantic choice of language when describing autism, as members of the autism and autistic community preferred to use *identity*-*first* language (autistic person), whereas professionals were more likely to use *person-first* language (person with autism). This study explored 803 e-learners’ responses from their comments across two massive open online courses on autism education held between 2017 and 2019. Learners agreed that autistic individuals should guide others on which terminology to use when describing autism, and although *identity-first* language acknowledges autism as part of an individual’s identity, it can also conjure up negative stereotypes and be stigmatising. Although family, friends and professionals highlighted that the diagnostic label is a way to facilitate understanding across stakeholder groups and help autistic individuals gain access to support, autistic self-advocates found the process of disclosing autism as a form of disability to conflict with their sense of identity, and broader terms such as ‘autism spectrum’ failed to capture individual strengths and weaknesses. Semantic language choices may matter less as long as the person’s difficulties are clearly acknowledged, with adaptations made to meet their specific needs. Adding to a growing body of literature on terminology use in autism research and practice, we highlight that language used when describing autism should follow the autistic individual’s lead, with the primary focus on communicating an individual’s strengths and difficulties, to foster a sense of positive autism identity and inclusivity, and enable access to appropriate support.

‘What’s in a name? That which we call a rose

By any other name would smell just as sweet’

(Shakespeare, trans. 2006, 2.2.43-44)

There has been an increasingly prominent debate among autistic self-advocates, family and friends, and professionals about the use of *person-first* or *identity-first* language when referring to autism (Bury et al., 2020; Kenny et al., 2016; Vivanti, 2020). Since the early 1990s, there has been a broadening of the autism spectrum, increasing public awareness and understanding, and the rising of the neurodiversity movement (Bagatell, 2010). Such transformations have contributed to division in how autism is perceived by members of the autistic and autism community^1^ as well as professionals, and consequently, there has been discourse around descriptors of ability and disability and diagnostic labels for autism. On one hand is the biomedical conceptualisation of ‘autism as disease’, with some suggesting that autism is a condition that can be ‘fixed’ (Rioux & Bach, 1994), and on the other hand is the counter-metaphor of ‘autism within neurodiversity’, with reasonable adjustments being made in the sociocultural environment to enable individuals to thrive by utilising their strengths (Bagatell, 2010; Bottema-Beutel et al., 2020; Broderick & Ne’eman, 2008; McDermott & Varenne, 1995). However, a consensus has yet to be reached across the multiple stakeholder groups (including professionals, family and friends of autistic individuals, and self-advocates) on the language that should be embraced when referring to autism across contexts. Kenny et al. (2016) helped to bring this important issue that is well-documented in the self-advocacy world (Brown, 2011; Kapp et al., 2013; Sinclair, 1993, 1999), to the attention of those in the wider academic circle of autism. This article aims to explore the public reception of the *person-first* (‘person with autism’) versus *identity-first* (‘autistic person’) debate elevated by Kenny et al. (2016) through an online discussion forum attended by different stakeholder groups that were part of a series of massive open online courses (MOOCs) on supporting autistic individuals.

Autism spectrum disorder (ASD) is characterised by social communication deficits and restricted and repetitive behaviours, activities and interests in the most recent Diagnostic and Statistical Manual of Mental Disorders, Fifth Edition (*DSM*-5; American Psychiatric Association, 2013). The conceptualisation of autism as a *disorder* within the medical model highlights that the symptoms induce functional impairment and affect an individual’s quality of life, and therefore symptoms should be eliminated in order to increase one’s quality of life (Baker, 2011; Kapp et al., 2013). However, the translation of the medical model based on physical ailments to developmental conditions such as autism has been challenged, as it does not acknowledge how society and environment may be constructed in a way that fails to meet an individual’s needs and thus resulting in behaviours that are perceived to be ‘deficits’, and also fails to include personal strengths (Baker, 2011; Bottema-Beutel et al., 2020). Nevertheless, there have been developments in the medical field to conceptualise restrictions of activity as socially imposed or caused by impairment from a social-relational perspective (Thomas, 2004), and to characterise the multidimensionality of disability, for example, the development of the International Classification of Functioning, Disability and Health (ICF) (Bölte et al., 2014).

The medical model of autism guides practitioners and parents to view autism as something that can be ameliorated and can be separated from the individual (Langan, 2011). This idea that autism does not reflect an intrinsic part of the individual is carried over from the use of *person-first* language in the disability literature (Foreman, 2005). *Person-first* language has been adopted in both the psychological and educational literature, as well as by governments and agencies (Halmari, 2011). Originating in the disability rights movement in the 1970s, and more specifically in the self-advocacy movement of people with intellectual and developmental disabilities (Wehmeyer et al., 2000), the phrase ‘a person with . . . ’ highlights that the individual is not defined by their condition but rather that it is just one speck in the constellation of characteristics that make up that particular individual (Vivanti, 2020). Some proponents of *person-first* language also view disability as a socially constructed concept that changes over time depending on the level of accommodation provided by society; therefore, each person should be valued as a person first rather than highlighting their disability (Snow, 2006), reflecting the social model of disability (Oliver, 2013). However, this notion that *person-first* language is less stigmatising has not gone unchallenged. It has been suggested that semantically presenting the condition after the person might draw people to pay more attention to this piece of information, and in using this language primarily to describe individuals with disabilities, especially children and those with the most stigmatising disabilities, it serves to emphasise a vulnerability and perpetuate stigmatising views (Gernsbacher, 2017; Halmari, 2011).

With the rise of the autism rights movement, the idea that autism forms part of an individual’s identity influenced a move towards *identity-first* language (Sinclair, 1999). Parents have also voiced the increased need for individual differences in neurological development to be viewed as part of human diversity, in contrast to the dichotomy of normal and abnormal demarcated by the medical model (Langan, 2011). In the same way of denying a societal role in the medical model of disability, extreme propositions of the social model of disability that deny the role of biological characteristics equally provide a single-dimensional account and may overlook the daily experiences and challenges relating to autism (Anastasiou & Kauffman, 2013). However, the general view among neurodiversity advocates is that deficits should be reconceptualised as part of individual differences and strengths should be celebrated (Broderick & Ne’eman, 2008; Kapp et al., 2013), while understanding autism as a disability requiring appropriate support (den Houting, 2019). A preference for *identity-first* language was associated with being autistic or awareness of the neurodiversity movement in the first study of autistic and non-autistic people’s preferences for *identity-first* or *person-first* language by Kapp and colleagues (2013). In this context, the need to adopt *identity-first* language among healthcare professionals and to formulate each individual’s strengths and difficulties as part of neurodiversity, has been emphasised (Nicolaidis, 2012).

However, the use of *identity-first* language has not been uniformly embraced across different stakeholder groups, though its use has fostered stronger communities that embraced the neurodiversity model (Shakes & Cashin, 2020). In the widely cited study by Kenny et al. (2016) which explored different stakeholder perspectives on preferred terminology when describing autism in the United Kingdom, the authors found that a wide range of terms were used across professionals, autistic self-advocates and their family and friends, with ‘autism’, ‘on the autism spectrum’ and ‘autism spectrum disorder’ being most commonly cited. Although the study found that professionals were more likely to use *person-first language*, and autistic adults and families were more likely to use *identity-first* language, the distinction was not clear-cut, with many participants expressing the need for flexibility in language use depending on the situational context, form of communication and audience. A more recent study in Australia found that individual preferences for *person-first* or *identity-first* language were also negative correlated, with the term ‘autistic’ being rated as both most and least preferred term, highlighting the controversies around its use (Bury et al., 2020). Similar to findings from Kenny et al. (2016), the term ‘on the autism spectrum’ was cited more commonly across the sample as the overall preferred terminology when factoring both most and least preferred rankings, further highlighting that there is not uniform support of *identity-first* language.

Two recent commentaries have addressed the controversial debate around the most accepted and respectable way to refer to autism in research. Robison (2019) highlighted the ‘nothing about us, without us’ mantra from the autistic and wider disability community, and referred to the importance of inclusion at both the research and clinical practice level. Vivanti (2020) emphasised that the semantic differences in *person-first* and *identity-first* language represent two different pathways to achieve the common goal of de-pathologising autism, to increase understanding and acceptance, suggesting the choice of language reflects an issue of multifinality. Vivanti (2020) also reflected that those who preferred *identity-first language* in the Kenny et al. (2016) report by no means represent the general consensus of all self-advocates and their family and friends. Like Robison (2019), Vivanti (2020) emphasised that the decision on language use should consider the preferences of participants that took part in each research study, to truly reflect the idea of inclusion and participatory research, rather than a simple linguistic choice.

Beyond the debate between *person-first* or *identity-first* language, identifying language has been further complicated by the changes in diagnostic labels proposed by the *DSM*-5 (American Psychiatric Association, 2013), where previously separate conditions including Asperger’s Syndrome and Pervasive Developmental Disorder – Not Otherwise Specified were collapsed under the umbrella of ASD. This dimensional view has brought into question whether the new diagnosis truly encompasses the range of presentations captured by the previous separate conditions, and whether such a conceptualisation is relatively narrower such that some individuals who had previously been diagnosed may no longer fall under the ASD umbrella (Volkmar & Reichow, 2013). Such changes have not only challenged the subgroup identities that autistic individuals may have formed prior to the introduction of ASD but also received mixed reception from education and healthcare professionals, with some concerned that broadening the spectrum may increase stigmatisation of the condition (Kite et al., 2013), as well as challenge the clinical utility of autism labels for communicating information among professionals (First et al., 2004; Ruiz Calzada et al., 2012). These concerns resonate parents’ perspectives that diagnostic labels (such as Asperger’s and Autism rather than ASD) support accuracy in people’s perception of their child’s needs, though this study found that parents did not show any preference for terminology as long as understanding can be established (Ruiz Calzada et al., 2012). Many parents recognised the advantages their children can have from learning about their diagnosis such as developing a sense of identity, ownership and empowerment, though they also feared that such a label might lead to increased stigmatisation their child might face at school and did not want them to be defined by the condition (Crane et al., 2019).

Notions of acceptance and understanding through diagnostic labels have also been found among young people who had Asperger’s Syndrome in mainstream secondary schools (Humphrey & Lewis, 2008). Some students perceived the label marked them to be different from their peers, and also can alter the lens through which other people perceived them at school, such that their social naïveté often made them into easy targets for bullying among peers (Humphrey & Lewis, 2008). This heightened level of self-doubt and need for acceptance by others during adolescence is somewhat in contrast to autistic adults, who are more accepting of their own differences, expressed a need to educate others about autism through self-advocacy, and challenged the ideas of fitting in and being normal as imposed by neurotypicals (Hurlbutt & Chalmers, 2002). Greater self-acceptance of autism has also been associated with lower depressive symptoms (Cage et al., 2018) and better self-esteem (Cooper et al., 2017), suggesting that autism identity can be an important protective factor against some mental health difficulties among autistic adults.

## Current study

There are differences in language use within and between multiple stakeholder groups (including professionals, family and friends of autistic individuals, and self-advocates). Preliminary research findings suggest that self-advocates as a group tend to prefer *identity-first* language. In light of the self-advocacy movement and the increasing policy drive for client-centred practice in the United Kingdom, combined with the fluidity of language use, it is of great interest to investigate the public response to the language debate. Previous studies investigating individual differences in language preference have been overtly advertised research studies that may have attracted a biased sample already aware of such debates who used the research study as a platform to express their personal opinions. The current article aims to utilise a new research context in order to develop the existing evidence-base on the *person-first* versus *identity-first* debate elevated by Kenny et al. (2016).

In the context of a broader issue around increasing autism awareness and education among the general public, this study uses discussion forum data from a series of MOOCs that educated the public on good education practice and the use of digital technology to support autistic children and young people. Specifically, issues around the use of language and terminology when referring to autism are taught by drawing upon findings from the Kenny et al. (2016) paper. The MOOCs provide a unique opportunity to explore language preferences from discussion forums that formed part of a broader autism education course, such that the audience included a range of self-advocates and their families and friends, as well as education and healthcare professionals who were interested in using evidence-based practice to support autistic children and young people, and may have been naïve to the language debate prior to taking part in this online course. The intention of this study is not to find an objectively ‘correct’ way to describe autism and establish a consensus across diverse stakeholder groups. Instead, our focus was to explore the interaction between social knowledge, personal belief and actions that guides an individual’s choice of language, thus highlighting potential similarities and differences in the intended and perceived meaning and purpose conveyed by various terminology when used by autistic advocates, their families and friends and professionals from multiple disciplines.

## Method

### MOOCs

This study used comments from discussion forums on two MOOCs held on the FutureLearn platform that were developed and run by the host university (see Appendix 1 for further information about the MOOCs). Each MOOC ran over the course of 4 weeks, where signup was free for any learners around the world through FutureLearn, and learners were able to post freely their thoughts and feedback throughout the MOOC via the comments section on each page and take part in the questions posed on specific topics across various discussion forums. All comments were monitored by a faculty member and a doctoral student in the field of autism, who regularly posed and answered questions to and from learners to foster an interactive online learning environment. Between 2017 and 2019, SMART-ASD had four runs, and Good Practice had two runs. Both MOOCs attracted a global audience. SMART-ASD reached a total of 6824 learners (3210 were active learners) from six continents and 142 countries, the majority were from Europe (72.22% of joiners and 73.24% of active learners). Good Practice reached a total of 5015 learners (2089 were active learners) from six continents and 61 countries, the majority were also from Europe (67.5% of joiners and 68.31% of active learners). More detailed breakdown by continent is shown in Table 1.

**Table 1.** Countries of origin for learners from both massive open online courses.

Continent	SMART-ASD (6824 joiners, 3210 active learners)			Good education (5015 joiners, 2089 active learners)		
	Countries (n)	Joiners (n, % total)	Active learners (n, % total)	Countries (n)	Joiners (n, % total)	Active learners (n, % total)
Africa	23	218 (3.19)	81 (2.52)	7	237 (4.73)	87 (4.16)
Asia	42	511 (7.49)	206 (6.42)	26	552 (11.01)	205 (9.81)
Australia	3	405 (5.93)	225 (7.01)	3	339 (6.76)	140 (6.70)
Europe	42	4928 (72.22)	2351 (73.24)	16	3385 (67.50)	1427 (68.31)
North America	22	474 (6.95)	227 (7.07)	4	331 (6.60)	168 (8.04)
South America	10	119 (1.74)	53 (1.65)	5	96 (1.91)	36 (1.72)
Unknown	–	169 (2.48)	67 (2.09)	–	75 (1.50)	26 (1.24)

### Data collection

Data for this study was gathered from one discussion forum in the Week 1 module of both MOOCs across all six runs between 2017 and 2019. Week 1 covered key concepts around autism and included a series of articles and resources that covered the symptoms, screening and diagnosis of autism, as well theories, strengths and differences in terminology when referring to autism. Given that the MOOCs were open to anyone who was interested in autism, rather than recruited from autism specialist fields, we were unable to assume any level of prior knowledge that participants had before taking part in the online course. Therefore, to ensure that all learners established a common level of knowledge on language and terminology used to describe autism, learners were provided with background information and findings from Kenny et al. (2016)’s research, which highlighted that although different terminologies are used to describe autism, the most popular terms were ‘on the autism spectrum’ and ‘autism’, though differences emerged between professionals who preferred ‘person with autism’ and autistic adults who preferred ‘autistic’. Learners were then asked to take part in the discussion forum entitled *Differences in terminology* to answer two questions: (a) *What names and terms do you use when talking about autism (e.g. Asperger’s, person with autism, autistic, ASD, etc)?* and (b) *How important do you think the issue of different term is?*.

Overall, 803 learners participated in this discussion forum across the MOOCs, and a breakdown of relative percentages of family/friend, self-advocate and professionals as indicated by learners during Week 1’s self-introduction are shown in Table 2. Given that the *Good Practice in Autism Education* MOOC was focused on inclusive education practice, it attracted a relatively larger proportion of professionals that made up the overall pool of learners who took part in the discussion forum. We were unable to gather more detailed demographic information regarding age, sex, and country of origin for participants that commented on the discussion forum as we were unable to trace their user profile via FutureLearn. In total, 1203 comments were gathered across all six runs of both MOOCs on the *Differences in terminology* discussion forum.

**Table 2.** Learner demographic from both massive open online courses.

	SMART-ASD (n, % of total: 428)	Good Practice (n, % of total: 375)	Total combined (n, % of MOOCs combined: 803)
Self-advocate	22 (5.14)	15 (4)	37 (4.61)
Family/Friend	191 (44.63)	59 (15.73)	250 (31.13)
Professional	172 (40.19)	271 (72.27)	443 (55.17)
No role indicated	43 (10.05)	30 (8)	73 (9.10)

### Ethical considerations

Similar to a recent study which gathered data from a large public online domain (Twitter) (Shakes & Cashin, 2020), the current data collection also took into ethical consideration the issues around confidentiality and valid consent in using comments from this public e-learning website (FutureLearn) by following guidance on Internet-mediated research from the British Psychological Society (2017). Upon signing up to FutureLearn, users are informed that any comments that they choose to share will be publicly available and may be shared with university partners of FutureLearn for research purposes. Data gathered were treated as sensitive and deidentified, as each learner was only recognised by a random identification code generated by the FutureLearn platform. Any potentially identifiable information included in the quotes selected for this article were manually removed and altered to protect participants’ anonymity. While participants are unable to actively consent or withdraw from research, they were able to edit their learner profile on FutureLearn platform in the unlikely event that they wanted to withdraw any quotes. Commenting on the ‘Differences in terminology’ discussion forum was completely voluntary. There were no rewards for commenting and no restrictions for learners who did not comment. Ethical approval for analysing anonymised comments from this public e-learning website were obtained from the university’s psychology ethics committee, and do not infringe upon FutureLearn’s copyright.

### Data analysis

Data were analysed using thematic analysis following the Braun and Clarke (2006) method, with a focus on the semantic content of the data. Rather than splitting the data into different learner groups based on relationship to autism as done by Kenny et al. (2016), data analysis was conducted by treating the data set as a whole, in order to identify overarching themes across all learners and to highlight any nuanced differences between the learner groups within each code. Coding was completed by two independent raters (first and second author), and the third author consulted with the final coding framework (see Appendix 2 for further information on data analysis and coding process). Quantification of endorsement by participants from each stakeholder group (Table 4) was done *post hoc* to the development and selection of themes during thematic analyses. Endorsement was coded in a binary sense, based on whether the participant referred to the idea expressed by each theme within their comment (1) or not (0). Therefore, the number provided can be interpreted as a total headcount of participants from each stakeholder group who endorsed each theme. The sole purpose for providing the quantitative comparison is to characterise potential differences in the extent to which each stakeholder group related to the specific themes, supplementing the narrative synthesis of the thematic analysis outlined below. The quantification does not bear any significance on the relevant importance or ranking of the themes, as this is advised against by the thematic analysis approach outlined by Braun and Clarke (2006).

## Results

The total number of learners that expressed a preference towards certain terminologies are shown in Table 3. Both self-advocates and family/friends showed a clear preference towards identity-first language by embracing the term *autistic*. Professionals showed a more evenly distributed terminology preference across *person with autism, on the autism spectrum, autistic* and *ASD.* It should be noted that a large number of learners also expressed no clear preferences towards any terminology used.

**Table 3.** Preferred terminology expressed by learners across both massive open online courses.

Terminology	Self-advocates (*n* = 37)	Family/friends (*n* = 250)	Professionals (*n* = 443)
Autism	2	20	34
Has autism	2	33	15
Person with autism	7	24	90
On the autism spectrum	3	34	91
Autistic	20	87	69
ASD	3	44	73
ASC	1	8	13
High functioning autism/Asperger’s	6	16	13
Other	2	17	10
No preference indicated	5	49	133

ASD: autism spectrum disorder; ASC: autism spectrum condition.

We identified four overarching themes across the data set: perceptions and understanding of terminology, purpose of terminology, choosing terminology and person matters most. To give relative comparisons of different stakeholder groups’ voices, we have provided relative breakdown of endorsement and selected quotes from each learner group in Table 4. Quotes were chosen to best illustrate a broad range of stakeholder perspectives within each theme. A summary of each of the four themes is provided below. Given that the data was analysed across all participants rather than within each stakeholder group, we outline the shared ideas expressed by learners from different stakeholder groups when describing each theme. Where unique ideas are identified for specific groups or where opinions differed across the groups within a theme, the voice of each stakeholder group is outlined to highlight such nuanced differences.

**Table 4.** Themes and codes endorsed by learners across both massive open online courses.

	SA (n, %)	FAM/FRI (n, %)	PROF (n, %)	N/A (n, %)	Quotes
Perceptions and understanding of terminology					
All encompassing	2 (5.56)	10 (27.78)	21 (58.33)	3 (8.33)	*SA*: I think a common misconception is thinking of the autistic spectrum as a straight line. It isn’t one, there are no ends. Someone can be ‘high functioning’ in some areas of their life, while being ‘low functioning’ in others. Many people assume. *Fam/Fri*: I prefer the phrase on the autism spectrum. To say autistic kind of narrows the wide differences there can be. *Prof*: I personally like the term autism, as it puts all the variations together, making it easier for professionals to explain to autistic people what they have and the best way to live with this, also it is a relieve for parents to get a good diagnosis.
All part of autism	3 (23.08)	5 (38.46)	4 (30.77)	1 (7.69)	*SA*: I have no idea what motivates some people to prefer the term Asperger’s. . . because the simple fact is that call it whatever they wish they still have a form of autism. Asperger’s Syndrome is still part of the autistic spectrum. *Fam/Fri*: I usually say that my son is autistic. It really doesn’t matter for me which term people use: autism, ASD, autism spectrum. . . for me all those term mean the same. *Prof*: I think there is a lack of awareness regarding Asperger’s and Autism, with people not realising that they fall under the same umbrella.
Awareness of new terminology	1 (2.44)	10 (24.39)	28 (68.29)	2 (4.88)	*SA*: I think it’s an really important issue between people using different terminology and people understanding the terms and the differences between them. *Fam/Fri*: I hadn’t previously realised that there were other options so had preferred to say ASD rather than the whole unanticipated name, mainly because I did not like the word disorder in association with my small child. *Prof*: It now seems to me that autistic people see their condition in a very different way to how professionals see it by the difference in terminology each use. Until reading these comments I would have also said this is a person with autism or the person with an illness but the autistic see autism as being what makes them themselves. So terminology is very important.
Changing terminology	5 (20.83)	8 (33.33)	10 (41.67)	1 (4.17)	*SA*: I was offered an Asperger’s diagnosis but I feel that the association with high cognitive ability and unusual skill sets is divisive rather than inclusive and serves to promote a form of snobbery that I feel is detrimental to the autistic community. The term served its purpose in broadening our thinking about autism as a spectrum but is now not needed. *Fam/Fri*: The acceptable terminology has changed a lot since I started working in the field. I currently tend to refer to people as on the autistic spectrum and include people with additional learning difficulties and others often referred to as Asperger who manage to function with relatively few problems in modern society. *Proc*: In Italy people use different ‘titles’ as well, according to the environment they come from and the background. For instance a professional educator graduated in the 90s would call a child Autistic while the new professionals diversity The Asperger and the ASD child. I think the difference of terms is symptomatic of a variety of studies and approaches to Autism. If we think of the history of disability word itself it has started from handicap to disability to impairment. I believe this is a sign of something still developing.
Medicalising condition	5 (8.06)	20 (32.26)	26 (41.94)	11 (17.74)	*SA*: I don’t mind being called ‘autistic’ or ‘a person with autism’ but I don’t like the use of ‘Autism Spectrum Disorder’ because calling it a disorder to me makes it sound like there’s something wrong with that person when there isn’t. Call it a condition or a diagnosis, but it isn’t a disorder. *Fam/Fri*: Diagnosis is better from my point of view – disorder makes it sound like there is something wrong with the person, when really all they do is have a brain that thinks differently. *Prof*: medical terms are very strong at school, where many people lack knowledge on the topic and may unintentionally harm kids by treating them as if they were ill or ‘different’ and just autistic (instead of a person with many characteristics, amongst them autism).
Stigma	10 (9.71)	34 (33.01)	52 (50.49)	7 (6.80)	*SA*: The word ‘autistic’ conjures up many negative ideas in people’s minds due to what they have seen or read on media – mostly negative. *Fam/Fri*: The different terminology could lead to discrimination. . . referring to them as a person with ‘special need’ is more tolerable and decent. Reduce stigmatisation. *Prof*: This is very tricky because of the different ranges in severity of this ‘condition’. . . there are people who are mildly autistic and some who are severely autistic yet all are described as having a level of impairment. If you call someone autistic or on the autistic spectrum, are you describing or labelling?
Purpose of terminology					
Access to resources/support	2 (5.56)	13 (36.11)	20 (55.56)	0 (0)	*SA*: I’ve had to think about that a lot about whether I want to be classed as disabled or not. I have to say yes I’m disabled in some cases to access ‘reasonable adjustments’ but I don’t really believe that about myself, I just think of myself as ‘thinking differently to the majority’. There is such a gap in support services for children and adults who are autistic/Asperger’s and who don’t have a learning disability. *Fam/Fri*: The label is useful in terms of getting help at school and getting access to support out of school but it can also have drawbacks. My daughter developed mental health problems. . . and it took far too long to get appropriate help because health professionals assumed that her behaviour was associated with her autism (they had a very fixed idea of autistic behaviour) instead of it being a symptom of her mental health problems. *Prof*: The child feeling comfortable and confident using those words will then help them to request any adjustments needed as they age, e.g., when they get to secondary school or university there won’t always be a parent present and the child will need to feel confident stating their needs themselves. If terminology is hidden from them, it’ll make it far harder for them to explain what they need!
Accuracy in description	9 (17.65)	21 (41.18)	18 (35.29)	3 (5.88)	*SA*: To me, the phrase ‘on the spectrum’ sounds somewhat distant and like the speaker is trying to avoid using words such as ‘autism’ and ‘autistic’. Personally, I say I am autistic because I find it generally comes across as the most straightforward. *Fam/Fri*: I personally feel that the name is secondary and much less important than creating awareness about Autism. . . we often feel that it would be nice if we had more accurate way to define the type of autism our son has. Also, because it covers such a range of variations, every person has heard a bit about Autism and quickly jump into recommending a course of action based on what they know. *Prof*: The actual diagnosis is usually used in reports, to talk to professionals who might feel that it helps them understand the child’s needs – but even then each child is unique in their presentation which also takes into account their personality, interests, skills etc.
Identity	17 (25)	29 (42.65)	16 (23.53)	6 (8.82)	*SA*: Being autistic is just a fact. Like left handed people . . . we are just different. Whole and complete as we are. By separating it in language you drive the myth that the autism can be separated and that is toxic to forming an identity and positive mental health. *Fam/Fri*: Having children on the spectrum and working with children and young people I recognise that how they choose to be identified is key to building a good relationship with them and everyone identifies in different ways. *Prof*: I really get why autistic people don’t like person with autism as I am dyslexic and dyspraxic they are part of who I am and the autistic adults I know see the fact they are autistic as part of who they are. . . after all as my friend would say you do not say I am a person with Englishness you would say I am English!
Choosing terminology					
Choice for individual	16 (6.27)	74 (29.02)	144 (56.47)	21 (8.24)	*SA*: Terminology should respect the wishes of the community concerned, in this case, autistic people. A number of surveys show that we prefer ‘autistic’ or ‘autistic person’, and largely reject ‘person with autism’. There’s no ‘person without autism’ behind the ‘person with autism’. Autism IS who you are, in the same way that you’re a man, woman, American etc. *Fam/Fri*: I’ve been listening to actually autistic adults who tend to prefer autistic. Therefore, I will tend to use that unless it is appropriate to ask a specific person what they prefer. As an neurotypical mum of an autistic daughter I want to learn from those autistic adults who are sharing their experiences. *Prof*: I think it would depend on the individual person though. If I worked with older children/young adults then they may wish me to use a term that they feel the most comfortable with and I think that how we have to make sure we included everyone equally by ensuring that we are taking views into consideration.
Familiarity (context dependent)	6 (4.69)	47 (36.72)	68 (53.13)	7 (5.47)	*SA*: I tell people that I have social autism or Asperger’s and they automatically know what that means, but out of the both of them I always say I have social autism as that is easier for me to say. *Fam/Fri*: I don’t have a preference for labels as such. I find myself using different terms to fit in with other people. When I’m in the school environment, I use ASD amongst the teachers and other professionals as 99% of the time they know what that is. When I speak to other parents and carers I tend to say ‘on the spectrum’ mainly because I think it’s up to the individual to know what that means even if only loosely. *Prof*: I use person first language in a professional setting and when I am teaching people about Autism. When I am working with the person with Autism I use their name. I am also aware that people with Autism I know and work with like to refer to themselves as ‘an autistic person’. I would use this term with them. I think it’s about being respectful and not just having ‘one’ way.
Cultural difference	0 (0)	13 (23.64)	36 (65.45)	6 (10.91)	*Fam/Fri*: Unfortunately, my country did not give very much attention to the autism until now. Today we started to open the centres for ASD and terminology that is used is very much similar to the one in the course but used only by professionals in the field. Very rarely, people who are diagnosed or have family members with diagnosis, use different terms for a certain diagnosis. I believe the reason for that may be lack of knowledge and understanding on the subject, so people decide to go with the terminology that is a bit easier to use and it is usually ‘I have autism’ or ‘My son is autistic’, for all disorders in this spectrum. *Prof*: In my country, health professionals tend to speak of high or low functioning autistics, even ones that question this division.
Flexibility	4 (4.21)	40 (42.11)	45 (47.37)	6 (6.32)	*SA*: I say autistic, mum says I am on the spectrum – it depends what you feel comfortable with. *Fam/Fri*: My son has been diagnosed with Autism Spectrum Disorder. Sometimes I say my son has autism. Sometimes I say he is autistic. Sometimes I will say he is on the spectrum. Personally, it really does not matter to me what terminology people use, as long as it’s with respect. It will be interesting to see if my son has any preference or issue with it as he gets older. *Prof*: I would probably use person with autism or someone who is autistic interchangeable, however I would of course be sensitive to the response I receive if talking to someone who has autism in case they prefer something else. I think this is important to make people with autism feel comfortable and help people identify with it and understand it but also helps others be aware as well.
Preference	21 (7.78)	97 (35.93)	127 (47.04)	25 (9.26)	*SA 1*: I’m in the process of being diagnosed with autism, but will refer to myself as autistic if it turns out that the diagnosis is true. I follow a few people (adults) on Twitter who refer to themselves as autistic too. *SA 2*: In my case I prefer people to first and foremost acknowledge that they are a human being on the autism spectrum – autism doesn’t define who they are. *Fam/Fri*: Very interesting discussion. In a professional setting, I would use the term ASD. In a general setting, I strongly dislike referring to anyone by a label so I just avoid the conversation honestly unless I am asked. *Prof*: Personally I use terms as ‘autistic’ and ‘spectrum’, especially because it helps to mould the language to subtract the negative connotation off the diagnosis and help the people around them to focus more on how to help and less on the fact that they have autism. On the other hand I try to avoid enclosing the ASC as ‘Asperger and Autism’ because it tends to make people believe they are only two ways in which Autism can be found, which takes away the importance of specific needs that every autistic child has.
Professional vs. community	1 (1.12)	20 (22.47)	65 (73.03)	3 (3.37)	*SA*: I use the term ‘human with autism’ or ‘disabled person’ because some professionals forget sometimes that they have a real person on the other side and not a dehumanised thing. So I think it’s very important to discuss the term how it’s called. *Fam/Fri*: In my very first week of nursing training, our instructor told us that, when we walked onto our first wards, we wouldn’t see ‘thirty-two orthopaedic conditions’, but that we’d see thirty-two ‘people who happen to have orthopaedic conditions’. I’ve always remembered that, so I prefer the term ‘person with autism’, as does my older son who has autism. Only yesterday, he told me that he’s a ‘person’ who has autism, and he’d like to be seen as a person first. *Prof*: Whilst professions are trying to keep their language PC, I believe that we must take into consideration what the individual prefers. After all, they are the focus. Anything which avoids negative labelling, and empowers the individual must be considered. . . We will never achieve global consensus on terminology, but I am hopeful that we will achieve it in relation to respect and inclusion.
Age difference	0 (0)	0 (0)	4 (80)	1 (20)	*Prof*: As I work with young children, most of them are not yet fully aware of their diagnosis. This means they haven’t yet got a preference as to how they are referred.
Person matters most					
Acceptance	0 (0)	6 (50)	6 (50)	0 (0)	*Fam/Fri*: My friends are very keen for people not to see autism as something that can and should be ‘cured’, but rather as an alternative neurological way of being that should be accepted as just that, non-neurotypical. I agree with my friends that acceptance is the most important thing. *Prof*: I tend to use the term ASD as I find parents in particular can be very scared by their associations with the word Autism. So using the term spectrum and talking to them about some children being in different places within this seems to help some parents accept that their child is autistic.
It doesn’t matter	0 (0)	10 (31.25)	21 (62.63)	1 (3.13)	*Fam/Fri*: My son refers to himself as ‘autistic’ he is NOT a person with autism and he has an ADHD brain. Apart from that: he informed me that all those squabbles about terminology just give the impression of activity without actually doing something solid. *Prof*: The terms are less important to me (I appreciate they may be massively important to the learner) than the individual’s strengths and areas for development. My biggest fear is people that focus on the terminology to guide generic support and not actually the strategies that could help the individual.
Neurodiversity	1 (4)	10 (40)	12 (48)	2 (8)	*SA*: On this course, I’ve discovered the term ‘neurotypical’ – I’ve spent most of my life trying NOT to be typical especially as far as my thinking goes and I encourage my students to act equally diversely. Navigating is difficult when there’s so many different descriptors. I’d prefer neruodiverse and leave it there. *Fam/Fri*: I think differences should be recognised, acknowledged and taught how to deal with as the person and as the people interacting with the person, but calling them a disorder makes me think something is bad, when what we have already read has shown that there are positive aspects of being autistic. *Prof*: I understand that we are all somewhere on the spectrum but the majority of us are positioned fairly close together so our differences/deficits don’t really stand out as being an issue, we can think of ourselves as normal simply because we are aligned with the majority population.
Whole person (understanding, not terminology)	9 (4.25)	75 (35.38)	111 (52.36)	17 (8.02)	*SA*: From my end, I would prefer using the term: ‘An individual on the autism spectrum’, as it does not make use of the word ‘disorder’ (which may harbour negative connotations, and may be interpreted wrongly by the community/society - #differentnotless), and it also puts emphasis on the word ‘individual’ and/or ‘person’. In my opinion, such emphasis aids the individual concerned, to be perceived as a person/as a human being/as an individual, who is not entirely defined by autism (as autism does make part of one’s identity, however IT IS NOT ENTIRELY one’s identity). *Fam/Fri*: One thing that is key is that although we feel the ‘label’ is helpful in terms of meeting the individual needs and for understanding, it is not helpful in other ways. The key for us is that they are who they are and their individual ASD is just part of who they are and makes them individual. *Prof*: What is important is that as a professional educator I spot the early signs, seek guidance and diagnosis and support the child as an individual not as a number on a chart or stereotype.

SA: self-advocate; FAM: family; FRI: friend; PROF: professional; ASD: autism spectrum disorder; PC: politically correct; ASC: autism spectrum condition; ADHD: attention deficit hyperactivity disorder.

Percentages represent the relative proportion of each specific code that was endorsed by different learner groups.

### Perceptions and understanding of terminology

Learners from all three stakeholder groups discussed that the ‘autism spectrum’ is a broad terminology that is all encompassing in terms of capturing a broad range of individual differences. The use of such an umbrella term was received with mixed opinions. Some autistic self-advocates felt that the term ‘autism spectrum’ can overshadow their uneven profile of strengths and weaknesses and does not adequately capture their personal identity. In contrast, other autistic self-advocates as well as family and friends embraced the idea that autism should be perceived as one continuum and having distinct subcategories within the diagnosis was not helpful. For example, the separation of Asperger’s syndrome from autism may elicit different perceptions about the strengths and difficulties an individual has based on stereotypes, which can be misleading. Such distinctions were perceived to be ‘divisive rather than inclusive’ and did not help foster a unified autistic community. Professionals resonated the ideas from both sides of the argument and also expressed a desire to work more closely together with both the autistic and autism community to establish clearer guidance on what the terminology conveys for each individual, in order to use the term more appropriately when communicating with professionals, the autistic individual and their family.

Learners from the autism community and professionals also commented on how the MOOC had introduced them to a wide range of terminology used to describe autism, but also educated them in thinking about how each term may be perceived differently by members of the autistic community. By reflecting upon the attitude and judgement that is conveyed by one’s choice of language, learners from both stakeholder groups commented on becoming more aware of how identity-first language are preferred and perceived to be more respectful by the autistic community, an issue that some learners were unaware of prior to taking part in the MOOCs. Learners also reflected on the changing terminology used to describe autism over time and across culture, drawing parallels to similar language changes observed for referring to disability, and anticipated language to continue to evolve over time.

Autistic self-advocates commented on how their own personal preference for terminology have also changed over time, with some actively choosing to use identity-first language after reading blog posts and interacting with other self-advocates, and more openly embracing autism as part of their identity. However, controversies around the use of *identity-first* language (namely the term ‘autistic’) also arose, as some self-advocates and professionals commented that the distinction between description and labelling (the latter is deemed to be more stigmatising) to be unclear when using such language, and it may conjure up popular stereotypes of autism among the general public based on media and press, which can be stigmatising.

Learners from all three stakeholder groups highlighted that certain terminology such as the use of the word ‘disorder’ can medicalise the condition, and using such terms can be detrimental by eliciting stigma in certain occupational contexts such as in schools, where it might enforce a sense of what is ‘normal’ and ‘abnormal’ that is unhelpful in terms of treating an individual based on their specific needs. Stigma was also commented on more broadly, with several ideas proposed on how to reduce stigma, such as moving beyond the autism diagnostic label altogether and specifying each child’s strengths and difficulties when communicating with and between professionals.

### Purpose of terminology

Learners from all stakeholder groups identified three main advantages underlying the purpose of terminology. First, individual preferences for terminology needed to take into account how best to access support, resources, and accommodations in society, all of which depend on a valid diagnosis being in place. Families and professionals highlighted that teaching children and young people how to disclose and use their diagnosis to their advantage can help them access resources outside of the home environment. However, there are challenges within both the process and outcomes from disclosing one’s diagnosis. In terms of the process of disclosure, autistic self-advocates commented on feeling uncomfortable that autism disclosure often fell under the umbrella of disability, as they do not perceive autism to be a disability. Some perceived this conflict in identity as a barrier to disclosing their diagnosis, despite understanding that disclosure is often the gateway to accessing resources and forms of support. In terms of the outcome of disclosure, sometimes the *right* support to meet individuals’ needs can be lacking and having a diagnosis such as autism can lead to diagnostic overshadowing, where other co-occurring mental health conditions can be overlooked by the medical professionals as they are often attributed to the primary autism and/or specific learning disability diagnosis.

Second, learners from all three stakeholder groups commented that beyond the semantic choice of language, there lies a much greater need to accurately communicate individual’s unique strengths and needs across different contexts and stakeholder groups. Although the diagnostic label might facilitate understanding of the common characteristics of autism, it is important to pay attention to individual needs, and use whatever term is necessary to accurately communicate an individual’s strengths and difficulties. One term that aroused controversies among autistic self-advocates was the use of Asperger’s syndrome, as some felt that this term more closely captured their sense of identity and accurately described that they do not have co-occurring learning disability. However, others felt that this term was divisive for members of the autistic community, was no longer relevant under the new *DSM*-5, and also failed to acknowledge the uneven profile in intellectual and adaptive functioning.

Finally, learners from all three stakeholder groups acknowledged how autism forms a core part of an individual’s identity, and therefore why there may be a preference towards *identity-first* language that might be seen as more respectful by the autistic community when describing autism. For autistic self-advocates, many expressed that they preferred using identity-first language rather than alternative phrases that can be perceived as ‘polite euphemisms’ when describing themselves, which can be more stigmatising. For parents and family members of autistic children and young people, although many expressed that being autistic is just a different state of being, conflicting opinions arose when thinking about how to describe a young autistic child. Some parents felt that by embracing identity-first language from a young age, they can help their child build a positive image of autism as an important part of their identity. However, other parents preferred using other phrases to describe their child’s difficulties to avoid labelling them from a young age, and to give the child time to grow up and adopt a term that they feel most comfortable with when describing themselves. For professionals, many commented that language not only is used to communicate an individual’s strengths and weaknesses, but also describes the person’s identity, and the latter should be carefully considered when choosing language to describe an individual.

### Choosing terminology

Learners from all three stakeholder groups recognised that choices on language use should be guided by the individual in question, respecting the terms that they are comfortable with and prefer to use. Language primarily serves the purpose of communication, and learners reflected on how they often use a term that is familiar to the audience they are communicating with, as well as use language with a degree of flexibility. A contrast was clearly delineated by both family/friends and professionals who would often use *person-first* language or ASD when communicating with other professionals but would prefer to use *identity-first* language or follow the choice of the individual when communicating more informally with other stakeholder groups. Cultural differences were raised by families and friends as well as professionals, with some countries using more medicalised language when referring to clinical diagnosis, sometimes still divided autism into different subcategories (such as high and low functioning), due to lack of awareness on the different terms used to describe autism.

There was a wide range of preferences for terminology, suggesting that there is no unified consensus on what term should be used, even within each stakeholder group. For example, some self-advocates commented that they preferred *person-first* language because they felt that they were not defined by their autism, whereas others felt more comfortable using *identity-first* language as this has been advocated as the preferred choice by the online autistic community. It is clear from many of the comments that the language preference for many professionals, family and friends are shaped by their experiences of interacting with autistic individuals, rather than purely theoretically informed or based on personal preference. The shared underlying message across stakeholder groups was to choose a term that they felt to be respectful, nonstigmatising, and inclusive for the autistic individual in question. All three stakeholder groups highlighted that when uncertain of which terminology to use, the default option should be to actively seek out the preference of the autistic individual and/or their family. In the cases of children and young people who may not have developed a preference yet, stakeholders should be mindful of the language they use and frequently check in with the child/young person to see what language they feel comfortable with.

### Person matters most

All three stakeholder groups emphasised that beyond the debate around semantic language choice, it is more important to foster acceptance of individual differences and embrace a model of neurodiversity, rather than medicalising and pathologising autism as a disorder. In fact, some worried that such fervent discussions around language use might draw the public’s attention away from acceptance and understanding and that the latter is what should warrant a discussion, and not the former. Celebrating each individual as they are, to identify their strengths and recognise their difficulties, to ensure that they receive tailored support based on their needs is the utmost important message, and any ‘label’ should not lose sight of this final goal. The person matters the most, not terminology or language.

## Discussion

This study explored learners’ response to language and terminology used to describe autism cross six runs of two MOOCs on autism education via an e-learning platform. Our findings showed some consistency with Kenny et al. (2016) findings, whereby self-advocates and family/friends of autistic individuals showed a stronger preference for using *identity-first* language, and professionals showed a more widely distributed preference for both *person-first* language (‘person with autism’), *identity-first* language (‘autistic’), medical terminology (‘ASD’), and neutral terminology (‘on the autism spectrum’). Although comments revealed individual differences in preferred terminology, learners shared a common understanding that one should always prioritise autistic individuals’ choice of terminology and that different preferences used across contexts are to facilitate what one believes to be a shared understanding of an individual’s strengths and difficulties depending on the audience. Therefore, similar to Vivanti (2020) and Robison (2019)’s commentaries, there is no one-size-fits-all rule in terms of what is the most acceptable way of describing autism, and respecting the individual in question is the most important factor to consider beyond semantic choice.

With regards to the choice of language, the different stakeholder groups expressed the need to accurately communicate to others an individual’s strengths and weaknesses in order to access resources and support. In this context, all three stakeholder groups often resort to using more medicalised language that is otherwise less preferred in more informal and social contexts when describing autism, with the belief that such terminologies will help establish mutual understanding when interacting with or between professionals. For some autistic self-advocates, the use of words such as ‘disability’ and ‘disorder’ when describing themselves in order to access support stood in contrast to their self-identity and led to further barriers in their willingness to disclose, an issue that was overlooked by families, friends and professionals who described that children and young people should be encouraged to disclose their diagnosis in order to access support outside of the home.

Our findings highlight that it is important for families and professionals to recognise how language used during the disclosure process may cause conflict with autistic children and young people’s identity, and to carefully consider how best to take a person-centred approach and accurately describe one’s strengths and weaknesses in a way that aligns with the young person’s identity (Riccio et al., 2019). Using language that accurately describes an individual’s strengths and weaknesses is especially important as in some cases, the lack of awareness of individual differences in autism presentation among professionals can lead to issues such as diagnostic overshadowing, where co-occurring issues (such as symptoms of anxiety or low mood) may be misattributed by professionals as part of the primary autism diagnosis. Therefore, there seems to be communication barriers across stakeholder groups in formal healthcare and education settings when accessing the right types of support and services for autistic individuals.

### Implications for practice and research

The lack of understanding of individual differences in autism among professionals beyond the diagnostic label has been identified as an issue that contributes towards inequality in healthcare access for autistic children, young people and adults (Bruder et al., 2012; Kuhlthau et al., 2015; Nicolaidis et al., 2015; Zerbo et al., 2015). In a report that examined autistic adults’ experiences of communicating with healthcare professionals, many expressed frustrations that disclosing their autism diagnosis often translated into incorrect assumptions that clinicians held about their personal difficulties as well as misattributing non-autism-related behaviours as part of their primary diagnosis, and they often had to challenge such biases held by healthcare professionals (Nicolaidis et al., 2015). Therefore, it begs the question of whether using medicalising terminology and diagnostic labels does actually establish a mutual ground for understanding across stakeholder groups, or whether it is associated with unhelpful stereotypes that hinders the ability to support the autistic individual as a whole. Clinicians who have a poor understanding of individual differences in autism, and lack acknowledgement of the high rates of co-occurring mental and physical health conditions alongside autism (Hollocks et al., 2019; Warner et al., 2018), might be especially prone to diagnostic overshadowing, and such misattribution of co-occurring symptoms can further create healthcare access inequalities for additional physical and mental health support services and impact on an autistic individual’s quality of life. In addition, taking into account recent evidence supporting social camouflaging and masking behaviours reported by many autistic individuals in social contexts (Dean et al., 2017; Hull et al., 2019; Lai et al., 2017), it is even more important to ensure that professionals are aware that there is no single presentation of autism as demarcated by the *DSM*-5 diagnostic criteria (American Psychiatric Association, 2013), so that they can look beyond the label and tailor their care to accommodate the specific needs of each individual.

Similarly, in education settings, having a good understanding of autism knowledge is an important predictor of inclusive practice to foster learning in different education systems (Baglieri & Shapiro, 2012; Segall, 2008; Segall & Campbell, 2012). Recent research showed that in the United Kingdom, a discrepancy was found between trainee teachers’ high scores on objective measures of autism knowledge, and self-reported low perceptions of one’s own subjective understanding of autism knowledge (Vincent & Ralston, 2020), which resonated prior findings that teachers’ knowledge of autism did not necessarily correlate with self-perceived competence in inclusive practice and supporting autistic students in the classroom (Busby et al., 2012; Talib & Paulson, 2015). Such findings highlight that beyond providing theoretically informed training on autism knowledge and awareness, teachers need more support around practical guidelines and how to implement evidence-based practice to foster true inclusion in the classroom and meet autistic children and young people’s education needs. Therefore, terminology and language choices do not necessarily translate into accessibility of the right types of support for autistic individuals, and more work needs to be done to address this gap in communication between semantic language and functional support across both healthcare and education settings. In cases where there may be a lack of preference from the autistic self-advocate, or for those who may feel uncomfortable endorsing either person-first or identity-first language due to fear of miscommunication, the more neutral term ‘on the autism spectrum’ may be a suitable alternative to adopt, as previous findings have found that this was endorsed across the different stakeholder groups (albeit with less enthusiasm) with less controversy (Bury et al., 2020; Kenny et al., 2016).

Despite appreciating the importance of discussing language and terminology, learners also expressed a concern that the arguments around language and terminology use might be somewhat distracting, drawing attention away from the more important issues in autism such as improving autism knowledge and acceptance, and adopting person-centred approach to address each individual’s strengths and weaknesses. Autism researchers have continued to highlight the importance of engaging with members of the autistic and autism community in participatory research to identify future research directions that are most meaningful to them, in accordance with the ‘nothing about us without us’ principle from the autistic community (Brosnan et al., 2016, 2017; Fletcher-Watson et al., 2019; Parsons et al., 2020; Pellicano et al., 2014a, 2014b; Pellicano & Stears, 2011). Kenny et al. (2016)’s report has brought forth many discussions and research around terminology and language use across stakeholder groups and cultures, and has raised awareness around neurodiversity, autism identity, listening to and respecting the opinions of autistic individuals, and also highlighted how semantic choices can generate miscommunication and misunderstanding across stakeholder groups (Robison, 2019; Vivanti, 2020). It is these latter issues that should continue to be discussed beyond the limited scope of language and terminology choice. Fletcher-Watson et al. (2019) discussed in their recent paper entitled ‘*Making the future together: Shaping autism research through meaningful participation*’ that participatory research should endorse *partnership* with autistic people, *engagement* with the autism community, and *consultation* with individuals and organisations. To continue such dialogues across stakeholder groups relies on ‘*an open acknowledgement of the inevitability of disagreement*’ (Pellicano & Stears, 2011, p. 277). Perhaps the discussions around language use alludes to a much greater issue of how stakeholder groups can overcome such communication barriers and reach a mutual understanding on how to best support autistic individuals in society, and one should not lose sight of this overarching goal along the journey.

## Study limitations and strengths

Similar to Bury et al. (2020), one limitation of this study is that the method of gathering data via online discussion forums did not allow us to collect more in-depth demographic information on the learners. Therefore, we are unable to comment on how representative the current sample may be when reflecting opinions from different stakeholder groups. Given that the nature of the MOOCs focused on evidence-based practice in technology use and inclusive education for autistic children and young people, the MOOCs attracted mostly families and parents of autistic children, as well as teachers and other professionals who work with autistic children and young people, rather than autistic self-advocates themselves. The under-representation of autistic self-advocates is therefore a major study limitation, and it should be noted that autistic self-advocates who took part in either MOOC were more likely to be adults themselves and may not necessarily reflect the opinions of autistic children and young people themselves. Autistic adults may be more likely to have a more secure and well-established sense of identity based on personal experiences that both informs and is reflected by their choice of terminology. In contrast, autistic children and young people may still be either developing their personal preference or be influenced by how autism is described by family members and professionals around them, and their voices may differ from the autistic self-advocates captured in this study and remains to be explored.

Furthermore, given that the majority of the participants in the autism community stakeholder group were parents, grandparents, or extended family members of autistic children and young people, with only a few who claimed to be family friends of autistic individuals, we chose to analyse the comments from both family and friends together to give a more meaningful representation of the range of opinions expressed by members of the autism community, much akin to the analysis completed by Kenny et al. (2016). However, it should be acknowledged that such combined analyses may be insufficient in highlighting more nuanced differences in opinions across family versus friends of autistic individuals and is a limitation for both our study and that of Kenny et al. (2016). Future studies should seek to employ both larger and more representative samples of each subgroup within the autism community to assess more nuanced differences in their opinions and preferences.

One strength of our study was that the MOOCs were not directly focused on autism and language use per se but had a broader focus on autism in education and use of technology. The broader focus attracted a more diverse group of participants who may not have necessarily encountered the issue around language and terminology use in autism prior to participating in the MOOC. Although we were unable to assess participants’ baseline level of autism knowledge and familiarity with the different terminology used to describe autism, we were encouraged to see that many respondents replied in the comments section had highlighted how the MOOC brought to their attention the meanings conveyed by different terminology use in autism and elicited much personal reflection and shaped their understanding of their personal preferences. Therefore, a potential strength of the study is in capturing how a more diverse audience respond to the arguments presented around language use in autism, who may have otherwise not taken part in a research study that directly targeted people’s perceptions of different autism-related terminologies. Nonetheless, there may still be an element of selection bias given that only a fraction of the overall active learners participated in the online discussion forums (perhaps only representing learners with stronger opinions), and therefore, the data presented may not fully represent the full range of opinions within each stakeholder group in the wider community.

## Conclusion

In conclusion, our study adds to a growing body of literature that have examined the most preferred and respectful discourse when describing autism. By examining a large group of online learners’ reflections when presented with information on both side of the argument surrounding the use of language and terminology when describing autism, we found that there was no uniformity across different stakeholder groups on one single preferred term to use when describing autism. The consensus across stakeholder groups is that the autistic individual’s opinion should always be respected and prioritised, and language should strive to accurately convey individual’s unique strengths and weaknesses by adopting a person-centred approach. Beyond the discrepancies in language preference lie broader issues on how to bridge communication barriers across stakeholder groups and to engage autistic and autism community more meaningfully to inform research and practice.

## Appendix 1

### Massive open online course (further information)

The first MOOC was entitled *SMART-ASD: Matching autistic people with technology resources* and had a particular focus on educating learners on different types of technology available and tools to help learners critically evaluate each form of technology’s usability and appropriateness for any particular autistic individual. The second MOOC was entitled *Good Practice in Autism Education* and had a particular focus on educating learners on different types of school systems and structures in the United Kingdom for autism education, as well as how to foster inclusivity and use evidence-based practice in autism education. Both MOOCs were advertised via social media and the host university’s website to attract a global audience who may be interested in learning how to adopt evidence-based practice to best support autistic individuals in home and school settings. Given the nature and focus of the two MOOCs, *SMART-ASD* attracted many parents and family members of autistic children as well as teachers and professionals working with autistic children and young people, who are interested in learning more about how to best integrate technology into children’s daily lives at home. In contrast, the *Good Practice* MOOC attracted a larger number of professionals working in special education setting who are interested in how to adopt evidence-based practice to foster inclusivity in school settings. The adverts did not actively recruit for autistic self-advocates to take part in the MOOCs (i.e. self-advocates were equally welcome to participate in either MOOC as any other members of the wider autism community, or anyone else form the general public who were interested in learning more about autism and evidence-based practice).

## Appendix 2

### Data analysis

Given that this study aimed to understand learners’ responses to the use of terminology and language when describing autism, we adopted a critical realist position when analysing our data. We provided participants with some background information on the different opinions on language and terminology use when describing autism in the autistic and autism community (the empirical). We then invited participants to take part in the discussion forum where they expressed their current beliefs about autism terminology and use of language when describing autism in their daily lives (the actual). We invited participants to also share their reflections on how they may now perceive different terminologies and choose their language after reviewing research examining perspectives from multiple stakeholder groups (the real).

For coding, the first and second author familiarised themselves with the entire data set. Each independently coded 50% of the data and noted down a short sentence describing the meaning of each code. The authors met to discuss the coding framework and examined the quotes that were captured under each code to ensure that the description provided an accurate summary of the data within each code. Codes that showed a high level of similarity were merged and provided with a new code name to form the final coding framework. When discrepancies arose, such as when a code was identified by one of the two coders only, a group discussion with the third author helped to establish whether the data summarised under such codes can be collated with the existing framework, or whether a new code should be added. After agreeing upon and using the final coding framework to independently recode 50% of the entire data set each, the first and second author cross-examined a randomly selected 10% of the comments from each other’s data set to check for coding validity, and inter-rater reliability reached 88.43%. After coding, all three authors met to discuss how codes may best relate to each other in order to generate themes and cross examined select quotes from the different themes to check that each theme summarised a unique aspect of the data set and did not overlap with others. Each coder then reviewed their coded data set to select quotes that represented each stakeholder group’s perspective within the different codes and themes, paying special attention to highlight any nuanced differences between stakeholder groups.

## Notes

1. Autistic community include autistic self-advocates. Autism community includes family and friends of autistic individuals.
